# Long‐term stability assessment of a 4D tumor tracking system integrated into a gimbaled linear accelerator

**DOI:** 10.1120/jacmp.v16i5.5679

**Published:** 2015-09-08

**Authors:** Mami Akimoto, Mitsuhiro Nakamura, Yuki Miyabe, Nobutaka Mukumoto, Kenji Yokota, Takashi Mizowaki, Masahiro Hiraoka

**Affiliations:** ^1^ Department of Radiation Oncology and Image‐applied Therapy Graduate School of Medicine, Kyoto University Kyoto Japan

**Keywords:** Vero4DRT, dynamic tumor tracking, quality assurance, correlation model

## Abstract

We assessed long‐term stability of tracking accuracy using the Vero4DRT system. This metric was observed between September 2012 and March 2015. A programmable respiratory motion phantom, designed to move phantoms synchronously with respiratory surrogates, was used. The infrared (IR) markers moved in the anterior–posterior (AP) direction as respiratory surrogates, while a cube phantom with a steel ball at the center, representing the tumor, and with radiopaque markers around it moved in the superior–inferior (SI) direction with one‐dimensional (1D) sinusoidal patterns. A correlation model between the tumor and IR marker motion (4D model) was created from the training data obtained for 20 s just before beam delivery. The irradiation field was set to $3\times 3\;{\text{cm}}^{2}$ and 300 monitor units (MUs) of desired MV X‐ray beam were delivered. The gantry and ring angles were set to 0° and 45°, respectively. During beam delivery, the system recorded approximately 60 electronic portal imaging device (EPID) images. We analyzed: 1) the predictive accuracy of the 4D model ($E_{P}$), defined as the difference between the detected and predicted target positions during 4D model creation, and 2) the tracking accuracy ($E_{T}$), defined as the difference between the center of the steel ball and the MV X‐ray field on the EPID image. The median values of mean plus two standard deviations (SDs) for $E_{P}$ were 0.06, 0.35, and 0.06 mm in the left–right (LR), SI, and AP directions, respectively. The mean values of maximum deviation for $E_{T}$ were 0.38, 0.49, and 0.53 mm and the coefficients of variance (CV) were 0.16, 0.10, and 0.05 in lateral, longitudinal, and 2D directions, respectively. Consequently, the IR Tracking accuracy was consistent over a period of two years. Our proposed method assessed the overall tracking accuracy readily using real‐time EPID images, and proved to be a useful QA tool for dynamic tumor tracking with the Vero4DRT system.

PACS number: 87.59.‐e, 88.10.gc, 87.55.Qr

## I. INTRODUCTION

When treating thoracic and abdominal cancer patients, one of the most important concerns is intrafractional motion. Without motion management, intrafractional motion affects the accuracy of beam delivery in high‐precision radiotherapy,^1^, ^2^, ^3^ which may increase the rate of complications in the normal tissue surrounding the target.

Various methods have been proposed to reduce the impact of intrafractional motion during beam delivery including forced shallow‐breathing, breath holding, respiratory gating, and dynamic tumor tracking (DTT).^4^, ^5^ Recent interest has focused on DTT methods because of the ability to reduce the internal margin without an extension of the treatment time or burdening patients with breath holding. In clinical practice, various DTT methods, such as dynamic multileaf collimator (MLC),^(6)^ gimbaled head,^(7)^ robotic arm,^(8)^ and couch motion,^(9)^ have been developed.

The Vero4DRT system (Mitsubishi Heavy Industries, Ltd., Hiroshima, Japan, and BrainLAB AG, Feldkirchen, Germany) is capable of hybrid infrared (IR) marker‐based DTT (“IR Tracking”).^7^, ^10^, ^11^ The world's first application of IR Tracking with the Vero4DRT system was performed on a lung cancer patient at Kyoto University Hospital in September 2011.^(7)^ We have also treated liver and pancreatic cancers with the combination of IR Tracking and intensity‐modulated radiotherapy since June 2013.

IR Tracking can be performed by swinging the gimbaled X‐ray head; however, the weight of the gimbaled head is approximately 600 Kg.^12^, ^13^ Thus, IR Tracking may put additional stress on the gimbaled head, which may introduce the unexpected changes in machine performance.

The American Association of Physicists in Medicine (AAPM) TG142 report includes a recommendation for general quality assurance (QA) tests for medical accelerators.^(14)^ This report mentioned that machine parameters can deviate from their baseline values, measured at the time of acceptance and commissioning, for many reasons. There can be unexpected changes in machine performance due to machine malfunction, mechanical breakdown, physical accidents, or component failure. Additionally, there can be gradual changes as a result of aging of the machine components. These patterns of failure must be considered when establishing a periodic QA program. The AAPM TG76 report^(5)^ recommends addressing the calibration of the spatial relationship between the tracking coordinate system and the beam delivery coordinate system. However, the report makes no mention of general QA tests for DTT systems. In addition, although several investigators have reported fundamental performance assessments of the Vero4DRT,^10^, ^11^, ^15^, ^16^, ^17^, ^18^, ^19^, ^20^ there are no reports regarding general QA tests of IR Tracking, and no ‘gold standard’ methods for QA of the Vero4DRT system.

To perform IR Tracking safely and accurately, the long‐term stability of tracking system is clinically important. Thus, with this goal in mind, we performed the long‐term stability assessment of our IR Tracking system using the one‐dimensional (1D) motion platform and electronic portal imaging device (EPID) images.

## II. MATERIALS AND METHODS

### A. Description of the Vero4DRT system

**Figure 1:**
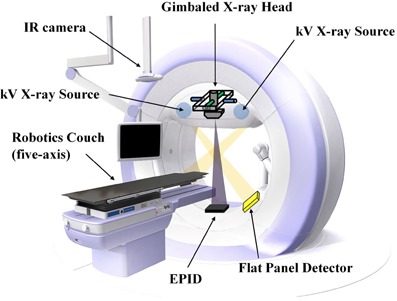
Appearance of the Vero4DRT system.

**Figure 2:**
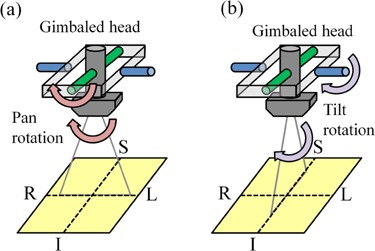
Definition of (a) pan and (b) tilt rotation for gimbaled head.

The Vero4DRT system has several special features that differ from other radiotherapy units. This system has an orthogonal kV X‐ray imaging subsystem, an EPID system, and a gimbaled X‐ray head with a compact 6 MV C‐band klystron‐based accelerator and a system‐specific multileaf collimator (MLC) in an O‐ring.^12^, ^13^ The five‐axis robotic treatment couch has high precision, of 0.1 mm and 0.1°, and an IR camera is attached to the ceiling of the treatment room (Fig. 1). The gantry can be rotated by ± 185° along an O‐shaped guide lane at a nominal maximum speed of 7°/s, and the O‐ring can be rotated by ± 60° around its vertical axis at a nominal maximum speed of 3°/s. The gimbaled X‐ray head can swing along the two orthogonal gimbals (pan and tilt rotations) up to ±2.5°, with a maximum rotational speed of 9°/s. The definitions of pan and tilt rotations are shown in Fig. 2. Pan refers to lateral angles and tilt refers to longitudinal angles. The gimbaled X‐ray head can swing the beam up to ±41.9 mm, with a maximum speed of 152 mm/s in each direction from the isocenter on the isocenter plane, perpendicular to the beam axis. As a result of this features, the MV X‐ray head can reposition the MV beam efficiently around the isocenter for tumor‐motion compensation. The positional accuracy of the gimbaled MV X‐ray head is <0.1 mm at any point within the $40\times 40\;{\text{mm}}^{2}$ area around the isocenter. The MLC is of single‐focus design, has 30 pairs of leaves each projecting 5 mm width at the isocenter, and produces a maximum field size of $150\times 150\;{\text{mm}}^{2}$.^(19)^ The maximum dose rate is set at 500 monitor units (MUs)/min. The EPID system has a pixel size of $0.18\times 0.18\;{\text{mm}}^{2}$ at the isocenter level and a matrix size of 1024×1024 pixels. The orthogonal kV X‐ray imaging subsystem consists of two sets of X‐ray tubes and flat‐panel detectors (FPDs) with a spatial resolution of 0.2 mm at the isocenter level, which can acquire radiographic, fluoroscopic, and cone‐beam computed tomography images.^(18)^ Additionally, the IR camera monitors the IR markers on the abdominal wall of the patients with an accuracy of better than 0.3 mm, which can detect the IR marker positions synchronously with fluoroscopic images.

### B. Phantom study

**Figure 3:**
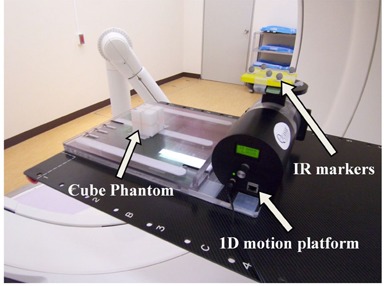
Diagram of the experimental setup for investigating tracking accuracy.

Figure 3 shows a diagram of the experimental setup for measuring the tracking accuracy. The QUASAR Programmable Respiratory Motion Platform (Modus Medical Device Inc., London, ON, Canada), which is designed to move phantoms synchronously with respiratory surrogates, was used. The IR markers moved in the anterior–posterior (AP) direction as an external surrogate, while a cube phantom having a steel ball at the center, as a tumor, and radiopaque markers implanted around it moved in the superior–inferior (SI) direction for 1D sinusoidal patterns (amplitude [A]: ±10 mm, breathing cycle [T]: 6 s) synchronously with the IR markers. The cube phantom and IR marker motions were perfectly correlated.

Prior to beam delivery, the IR and implanted markers were monitored synchronously for 20 s with the IR camera and the orthogonal kV X‐ray imaging subsystem, respectively. The sampling time intervals were 16.7 ms for the IR camera and 80 or 160 ms for the orthogonal kV X‐ray imaging subsystem. The sampling rate of the orthogonal kV X‐ray imaging subsystem changed automatically, depending on the speed of IR marker motion. Using the acquired training data, a 4D model was created to establish correlation between the 3D target positions indicated by the detected positions of implanted markers and the IR marker positions on the abdominal wall. The 4D model was expressed as a quadratic equation, involving IR marker positions and the speed of the IR marker motion.^(15)^ During beam delivery, the Vero4DRT system anticipates the future 3D target positions (predicted target position) from the IR marker positions and their velocity using the 4D model in real time, and then the gimbaled head tracks the moving target continuously. The irradiation field was set to $3\times 3\;{\text{cm}}^{2}$ and the 6 MV X‐ray beam was delivered for 300 MUs. To investigate the pan and tilt motion of the gimbaled head simultaneously, the gantry and ring angles were set to 0° and 45°, respectively. During beam delivery, the system recorded approximately 60 EPID images.

### C. Data analysis

**Figure 4:**
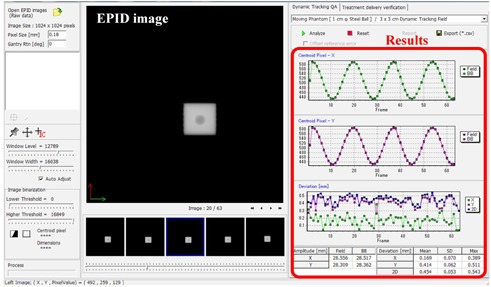
Screenshot of our in‐house software that can automatically detect the center of the steel ball and the center of the MV X‐ray field from EPID images. This software also calculates the deviations between the two centers.

We observed the tracking accuracy of the 4D tracking system between September 2012 and March 2015. We analyzed two metrics. First, the predictive accuracy ($E_{P}$) of the 4D model, defined as the difference between the detected and predicted target positions during 4D model creation. The mean plus two standard deviations (mean + 2 SDs) was calculated for $E_{P}$. Secondly, the tracking accuracy at isocenter level ($E_{T}$), defined as the difference between the center of the steel ball and the center of the irradiated field on the EPID image at the isocenter level (Fig. 3). The maximum errors of the pan, tilt, and 2D directions were calculated for $E_{T}$. The center of the steel ball and the center of the irradiated field were detected using our in‐house software (Fig. 4).^(21)^ This software automatically determined the centers based on threshold image processing and the given information of the radiation field size and the steel ball diameter. The analysis was performed each week when IR Tracking was scheduled. Otherwise, it was done once every month.

## III. RESULTS

**Figure 5:**
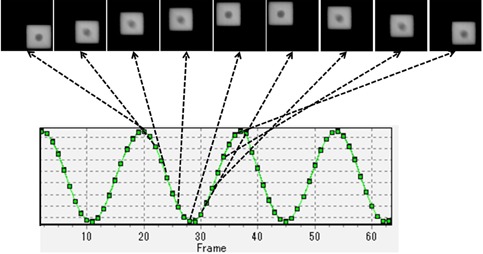
EPID images during IR Tracking. The gimbaled head can track the target using IR markers and the 4D model.

**Figure 6:**
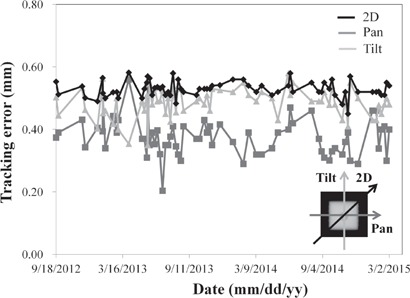
Maximum tracking errors in the pan, tilt, and 2D directions for each day.

Figure 5 shows the EPID images during IR Tracking. The median values of the mean +2 SD of $E_{P}$ were 0.06 (range, 0.00–0.12) mm, 0.35 (range, 0.13–1.07) mm, and 0.06 (range, 0.00–0.19) mm in the LR, SI, and AP directions, respectively. These results show that the Vero4DRT system was able to predict the target position from IR marker motion with high accuracy. The mean values of maximum $E_{T}$ were 0.38 (range, 0.20–0.56) mm, 0.49 (range, 0.35–0.58) mm, and 0.53 (range, 0.45–0.58) mm, and the CV values were 0.16, 0.10, and 0.05 in the pan, tilt, and 2D directions, respectively. Figure 6 shows the maximum value of $E_{T}$ in the pan, tilt, and 2D directions each day. It was found that the tracking errors in the pan, tilt, and 2D directions were almost the same every day. In addition, the difference between the tracking errors in the pan and tilt directions was with 0.32 mm. This QA test took approximately 10 min, including phantom setup of ∼3 min, 4D modeling and checking of ∼3 min, irradiation time of ∼1 min, and cleanup time of ∼3 min.

## IV. DISCUSSION

One important key issue in surrogate signal‐based DTT is the accuracy of the model predicting the internal target position based on the surrogate measurements.^(22)^ The results of the $E_{P}$ indicated that the Vero4DRT system was able to create a highly accurate correlation model between internal and external respiratory signals over a period of two years, as reported by other investigators.^10^, ^11^, ^15^, ^16^, ^17^

At our institution, the tolerance levels for daily QA of kV and MV X‐ray imaging are set at 0.5 and 0.2 mm, respectively. These errors might be included in $E_{T}$; however, the $E_{T}$ was up to 0.58 mm and the coefficient of variance was 0.05 in the 2D direction, which demonstrates that $E_{T}$ was significantly small and consistent over two years. In addition, the mean values of maximum $E_{T}$ were 0.38, 0.49, and 0.53 mm in the pan, tilt, and 2D directions. Some investigators have also assessed intrafractional tracking error in IR Tracking with a moving phantom. Depuydt et al.^(17)^ assessed the tracking error using a 1D moving phantom producing sinusoidal motion and the light field. They reported that the average values of the 90th percentile of the 2D tracking errors from the beam's eye view were 0.20, 0.22, and 0.54 mm in the pan, tilt, and 2D directions, similar to our results. Mukumoto et al.^(16)^ assessed the tracking error using a 3D moving phantom and a laser displacement gauge, and found that the 95th percentile tracking error ranged from 1.3 to 1.8 mm in the tracking state with a 1D sinusoidal pattern. They used a shorter breathing cycle (T: 2–4 s) for their analysis, which might have introduced the larger tracking error, compared with our results. Consequently, our proposed method can assess the overall tracking accuracy with a similar precision using MV X‐ray beams and EPID, which is useful for QA of IR Tracking.

The CyberKnife Robotic Radiosurgery platform (Accuracy Inc., Sunnyvale, CA) is another representative DTT system other than Vero4DRT.^(8)^ The Cyberknife compensates for tumor motion by moving a robotic arm based on the internal target position estimated from the movements of light‐emitting diodes on the chest, using a correlation model. The CyberKnife moves with more degrees of freedom than does the gimbaled linac. AAPM TG135^(23)^ recommends monthly checking of any unusual robot noises or vibrations associated with the Synchrony tracking system integrated into the CyberKnife. For the CyberKnife system, the geometrical relationship between the tracking system and the beam delivery system is verified via an end‐to‐end, dose delivery test using a specifically designed composite imaging and dosimetry phantom. The phantom is localized within the CyberKnife dose delivery system, and uses the imaging and tracking system and is irradiated with the planned dose. The delivered dose distribution, relative to the plan, reveals any systematic coalignment error in the tracking and delivery systems. If this alignment is compromised, the delivered dose will be shifted from its intended location in the phantom. It is important to develop a method for checking the error between the irradiated position and tumor position easily. Thus, the CyberKnife system had an original QA program.

As compared to the CyberKnife, the Vero4DRT system is a relatively novel 4D radiotherapy system with an integrated IR Tracking system. Solberg et al.^(24)^ have described a comprehensive commissioning study other than DTT for Vero4DRT system, and Depudty et al.^(25)^ have reported the imaging and treatment coordinate coincidence using a star shot test. They evaluated the mechanical performance of the Vero4DRT system; however, no systematic QA method for IR Tracking has been established to check the positioning accuracy of the tracking and irradiated coordinate system. Using our proposed QA method, one can check the overall tracking accuracy quite effectively. In addition, the pan and tilt motion of the gimbaled head can be checked simultaneously because the ring angles are set to 45°. This test takes only about 10 min.

## V. CONCLUSIONS

We estimated the long‐term stability of the tracking accuracy of the Vero4DRT system using the 1D motion platform and EPID images. From our results, the IR Tracking accuracy was significantly small and consistent over two years. Our proposed method can evaluate the overall tracking accuracy easily and directly using EPID images, and provides a useful QA tool for IR Tracking of the Vero4DRT system.

## ACKNOWLEDGMENTS

This research was supported in part by the Center of Innovation Program from Japan Science and Technology Agency, JST.
